# Synthetic exendin-4 disrupts responding to reward predictive incentive cues in male rats

**DOI:** 10.3389/fnbeh.2024.1363497

**Published:** 2024-03-14

**Authors:** Ken T. Wakabayashi, Ajay N. Baindur, Malte Feja, Mauricio Suarez, Karie Chen, Kimberly Bernosky-Smith, Caroline E. Bass

**Affiliations:** ^1^Neurocircuitry of Motivated Behavior Laboratory, Department of Psychology, University of Nebraska-Lincoln, Lincoln, NE, United States; ^2^Department of Pharmacology and Toxicology, Jacobs School of Medicine and Biomedical Sciences, University at Buffalo, State University of New York, Buffalo, NY, United States; ^3^Clinical and Research Institute on Addictions, University at Buffalo, State University of New York, Buffalo, NY, United States; ^4^Department of Physician Assistant Studies, Canisius College, Buffalo, NY, United States

**Keywords:** glucagon-like peptide-1, sucrose, incentive salience, pharmacokinetics, effective dose

## Abstract

Synthetic exendin-4 (EX4, exenatide), is a GLP-1 receptor agonist used clinically to treat glycemia in Type-2 diabetes mellitus. EX4 also promotes weight loss and alters food reward-seeking behaviors in part due to activation of GLP-1 receptors in the mesolimbic dopamine system. Evidence suggests that GLP-1 receptor activity can directly attenuate cue-induced reward seeking. Here, we tested the effects of EX4 (0.6, 1.2, and 2.4 μg/kg, i.p.) on incentive cue (IC) responding, using a task where rats emit a nosepoke response during an intermittent reward-predictive IC to obtain a sucrose reward. EX4 dose-dependently attenuated responding to ICs and increased the latencies to respond to the IC and enter the sucrose reward cup. Moreover, EX4 dose-dependently decreased the total number of active port nosepokes for every cue presented. There was no effect of EX4 on the number of reward cup entries per reward earned, a related reward-seeking metric with similar locomotor demand. There was a dose-dependent interaction between the EX4 dose and session time on the responding to ICs and nosepoke response latency. The interaction indicated that effects of EX4 at the beginning and end of the session differed by the dose of EX4, suggesting dose-dependent pharmacokinetic effects. EX4 had no effect on free sucrose consumption behavior (i.e., total volume consumed, bout size, number of bouts) within the range of total sucrose volumes obtainable during the IC task (~3.5 ml). However, when rats were given unrestricted access for 1 h, where rats obtained much larger total volumes of sucrose (~30 ml), we observed some dose-dependent EX4 effects on drinking behavior, including decreases in total volume consumed. Together, these findings suggest that activation of the GLP-1 receptor modulates the incentive properties of cues attributed with motivational significance.

## 1 Introduction

Synthetic exendin-4 peptides are glucagon-like peptide-1 (GLP-1) receptor agonists that have been approved to treat type 2 diabetes mellitus. Exenatide (a.k.a. EX4, also sold under names of Byetta® or Bydureon®) regulates glycemia by activating peripheral GLP-1 receptors (GLP-1Rs) in the intestinal tract, resulting in insulin release and increased gastric emptying time (Nauck, 2011). Centrally, GLP-1-releasing neurons in the nucleus tractus solitarius (NTS) of the dorsal medulla respond to afferent vagal stimuli involving feeding and project widely throughout the brain to modulate sucrose intake and satiety (Merchenthaler et al., 1999; Grill et al., 2007; Holst, 2007; Cawthon et al., 2023). In addition to projections to areas involved in homeostatic feeding, some NTS GLP-1 neurons innervate mesolimbic areas involved in regulating reward-seeking behaviors (Alhadeff et al., 2012). For example, systemic, intracerebroventricular (ICV), and targeted EX4 microinfusions into the mesolimbic structures, including the nucleus accumbens (NAc) and ventral tegmental area (VTA), decreased lever pressing for food rewards and decreased food intake when highly palatable foods and standard chow were available concurrently (Dickson et al., 2012; Yang et al., 2014). We have previously demonstrated that EX4 dose-dependently attenuated operant responding for a sweetened fat reward under both the Fixed Ratio 1 and Progressive Ratio schedules (FR1 and PR, respectively; Bernosky-Smith et al., 2016), with EX4 being more effective in reducing FR1 responding.

GLP-1R agonists also impact drug-seeking behaviors that do not directly involve nutritive homeostasis (for a systemic review see Brunchmann et al., 2019). For example, EX4 decreases both amphetamine-induced accumbal dopamine release and conditioned place preference (Egecioglu et al., 2013). ICV delivery of EX4 suppresses cocaine-induced phasic dopamine release in the NAc core, but not shell, with no effect on electrically stimulated release (Fortin and Roitman, 2017). Systemic delivery of EX4 blocks cue-induced reinstatement of cocaine-seeking behavior at doses that did not impact measures of food intake, an effect attenuated by delivery of an intra-VTA GLP-1R antagonist (Hernandez et al., 2018). Likewise, central administration of EX4 also attenuates cue-induced reinstatement of sucrose seeking and conditioned place preference for a high-fat food in rats (Ong et al., 2017). Thus, in addition to the primary effects of EX4 and other GLP-1R agonists on the homeostatic drive for food, there is evidence that these drugs also alter responses to reward-associated cues themselves. Further supporting this hypothesis, in diabetic humans GLP-1R agonists have been shown to decrease neuronal activation induced by food cues (e.g., pictures of palatable food, Farr et al., 2016; Ten Kulve et al., 2016).

Food- or drug-seeking is a multifaceted process involving multiple reward-related systems including those that process cues that have been repeatedly paired with reward (Kelley and Berridge, 2002; Fields et al., 2007; Meyer et al., 2016). To test the hypothesis that GLP-1R agonism disrupts the incentive salience (Berridge and Robinson, 1998) attributed to a reward predictive cue, we determined how EX4 impacts operant responding in a task heavily dependent on incentive cues (ICs). During this task, rats must nosepoke during a random, 8 s, intermittent audiovisual cue to receive a sucrose reward. Behavioral metrics indicative of the motivational strength of the IC and primary reinforcer were measured. We hypothesized that EX4 would decrease the incentive motivation associated with the IC as well as the reinforcing properties of sucrose, resulting in a dose-dependent decrease in responding to the number of ICs and increase the latency to respond to ICs and to enter the reward receptacle.

## 2 Materials and methods

### 2.1 Subjects

Male Long Evans rats weighing between ~280–300 grams were purchased from Envigo (Indianapolis, IN) and individually housed under a 12:12 h light–dark cycle, with lights on at 3:00 PM. Food and water were available *ad libitum*. While satiety levels were not directly controlled during the experiment, all rats were pretreated and behaviorally tested at the same time each day. Rats were weighed on a weekly basis. All procedures were reviewed and approved by the University at Buffalo Institutional Animal Care and Use Committee.

### 2.2 Reagents

A synthetic EX4 (American Peptide Company/Bachem Americas, Inc., Torrance, CA) was suspended in 0.9% saline at a 1 mg/ml stock solution and stored at −80 °C until the day of the experiment. EX4 stocks were thawed on ice and serially diluted with sterile saline to 0.6, 1.2 and 2.4 μg/ml solutions prepared daily prior to each experiment. Sucrose was diluted with water to 10% concentration and stored at 4 °C.

### 2.3 Behavioral apparatus

#### 2.3.1 Operant chambers

Operant chambers (Med-Associates, Georgia, VT) were housed in sound attenuation cubicles. Each chamber was equipped with illuminated nosepokes located on the left and right of a central liquid receptacle with an infrared entry detector. The liquid cup was fitted with an 18-G cannula attached to the bottom of the cup connected to a syringe mounted in a pump. During IC operant task sessions, a 10 ml syringe was used to deliver ~64 μl of 10% sucrose solution over 4 s, while a 30 ml syringe was used during free drinking sessions to deliver ~45 μl/s sucrose solution. A dim white house light and speaker were located on the wall opposite the nosepokes and reward cup.

#### 2.3.2 Locomotor activity chambers

Locomotor activity chambers (43.4 × 43.4 × 30.3 cm Med-Associates, Georgia, VT) equipped with arrays of infrared sensors to detect locomotion were housed in larger sound attenuating cubicles illuminated by a dim white houselight.

### 2.4 IC operant task training and testing

**Figure 1:**
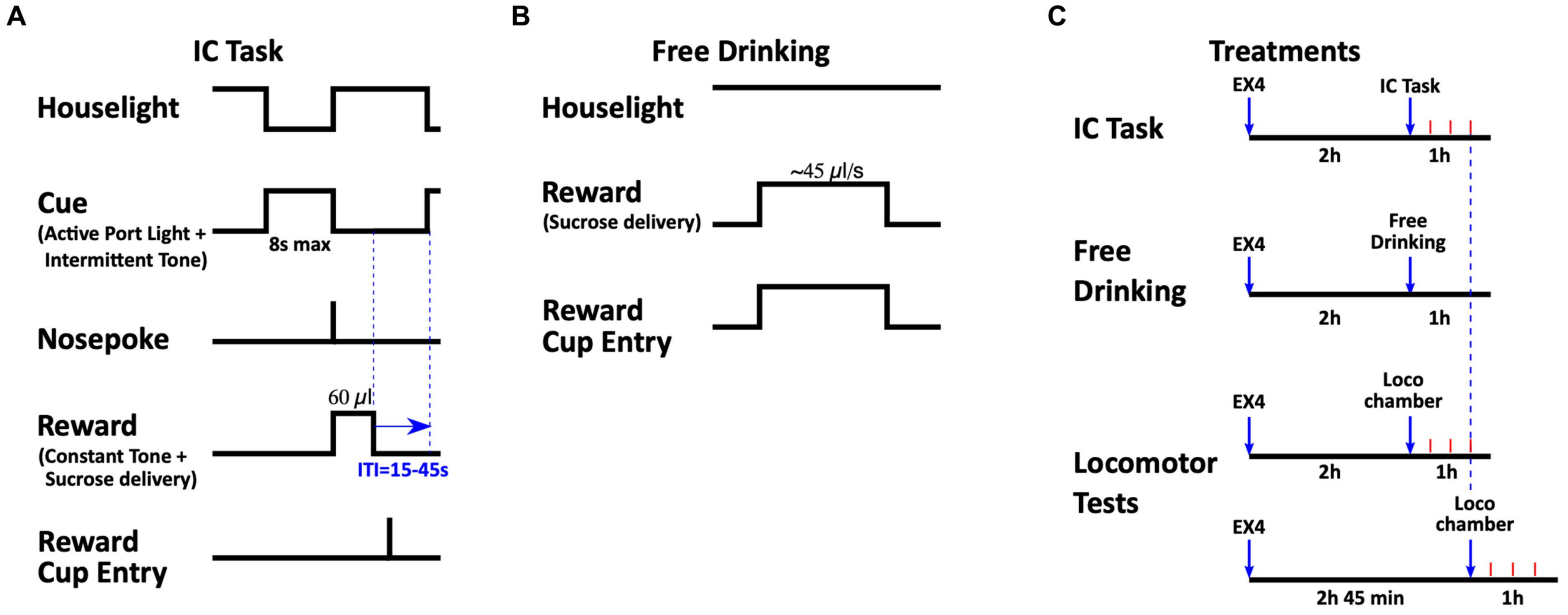
Schematics of the tasks and experimental design. **(A)** During the IC task, an intermittent audiovisual IC was presented on a variable interval 30 s schedule for a maximum duration of 8 s, during which a rat must have responded with a nosepoke in the active port. After a correct response, the IC was terminated and a distinct CS+ was presented while the sucrose reward was delivered in the reward cup. The entry into the reward receptacle was also recorded. **(B)** During the Free Drinking task, head entry into the reward cup triggered the continuous delivery of a sucrose reward. The houselight was illuminated throughout these sessions. **(C)** Timing of EX4 treatments. EX4 administration occurred 2 h before the beginning of the IC and free drinking sessions. EX4 treatments before testing locomotor activity occurred either 2 or 2 h 45 min before the beginning of the test, which corresponded to the first and last 15 min of the IC and free drinking sessions, respectively. Arrows represent EX4 administration and beginning of subsequent behavioral test. Red tick marks represent 15 min.

Rats (*n* = 11) were acclimated to the testing room for at least 30 min prior to being placed in an operant chamber. Each session lasted 1 h. The IC task is modified from other procedures described elsewhere (Wakabayashi et al., 2004, 2018, 2020; Yun et al., 2004; Ambroggi et al., 2011). Briefly, rats were first trained to nosepoke into the active nosepoke port to receive ~60 μl of 10% sucrose in the adjacent reward cup. Initially, a tone and light combination (consisting of an intermittent 2.9 kHz, ~80 dB tone with a 25/20 ms tone-on/off pulse, illumination of the active port while the houselight was turned off) was present during the entire session, except when the rat entered the port. Together, this combination of sound plus light stimuli comprised the IC. When the rat entered the active port, the audiovisual cue was terminated and the syringe pump was activated for 4 s, the houselight was illuminated and the tone changed from intermittent to constant; this comprised a conditioned stimulus (CS) that was distinct from the IC. There was no time-out period and the session terminated when the rat achieved 130 rewards or 1 h had elapsed. When the rats received at least 100 rewards for two consecutive days, the IC was decreased to 30-s total, and presented on a variable interval 30-s schedule in which inter-trial intervals between IC presentations were randomly selected from a Gaussian distribution (lower and upper limits 15 s and 45 s, respectively). Entry into the active port during the IC terminated it, followed by reward delivery under a continuous schedule of reinforcement (i.e., FR1), and presentation of the CS (Figure 1). Nosepokes into the port during the inter-trial interval, or during reward delivery, as well as entries into the inactive nosepoke, were recorded but had no consequences. Once rats achieved responding to 80% or more ICs during two consecutive sessions, the total IC length was set to 8 s, with ~100 trials during the 1-h session (Figure 1A). IC sessions were conducted at 10:30 AM, 6 days a week, and trained to a performance criterion of 80% responding to the ICs presented during the session. Stable acquisition of behavior (responding to 80% of ICs for two consecutive sessions) was required prior to drug testing.

Once performance criteria on the IC task was met, rats were challenged with EX4 in a Latin Square design. Rats were administered either saline, 0.6, 1.2, or 2.4 μg/kg of EX4, intraperitoneally (i.p.) 2 h prior to the start of the session. The doses and pretreatment times were based on our previous report (Bernosky-Smith et al., 2016) showing dose dependent EX4 effects on operant and locomotor behavior. At least three sessions with no pretreatment were allowed before the next challenge dose of EX4. The metrics analyzed included the: *total number of active nosepokes* made in the active nosepoke port during a session, the *number of inactive nosepokes* made in the inactive nosepoke port, *response ratio* (#active nosepokes resulting in a reward/#ICs), nosepoke *latencies (time; T)* to emit a rewarded nosepoke into the active port (*T*_nosepoke_-*T*_IC_) and enter the reward cup (*T*_cup entry_-*T_nosepoke_*), *nosepoke accuracy* (#rewarded active nosepokes/#total active nosepokes), *number of nosepokes in the active port for every IC presented* (#total active nosepokes/#IC), *number of rewards obtained* (#rewards), *number of cup entries per reward obtained* (#cup entries/#rewards), and *the ordinal number of the IC that was first rewarded*.

### 2.5 Free drinking task training and testing

New rats (*n* = 8) were tested in a free drinking session in the operant chambers identical to those used with the IC task, and configured as described previously (Wakabayashi et al., 2018, 2020). In this task, a head entry into the reward receptacle activated the pump, delivering sucrose continuously until the rat left the reward receptacle. Chambers were checked after each session to confirm all liquid had been consumed. Head entries in nosepoke ports had no programmed consequences, and the houselight remained lit for the entire duration of the test. Rats were trained for 3 consecutive days to acquire the task and to establish a baseline prior to commencing treatment. Rats were considered to have acquired the task once they consumed more than 0.8 ml of sucrose during the first 2 min of the free drinking session. Each session lasted 2 h and were run in two waves which began either at 8:30 AM or 11:00 AM. Once performance criterion was met, rats were challenged 2 h prior to the start of the session with EX4 in a Latin Square design identical to that of the IC task. Two days were allowed between challenge doses of EX4; rats were not run on intervening days. We analyzed three elements of free drinking behavior, including the *total volume consumed* by the rat derived from the total length of time the rat remained inside the reward receptacle and the flowrate of the pump, the *number of bouts* defined as each time the rat entered into the reward cup to consume reward, and the *bout size* defined as the average length of time the rat spent inside the reward receptacle per visit.

### 2.6 Locomotion testing

To determine whether the effects of EX4 during the IC task could be attributed to changes in locomotor behavior, a different group of rats (*n* = 7) were tested in a locomotor activity chamber. Rats were habituated to the locomotor chamber 1 h per day for 3 days prior to test sessions. Subsequently, rats were injected with either vehicle or 2.4 μg/kg EX4 (i.e., the dose yielding the largest change in behavior during the IC task) either 2 h, or 2 h 45 min before being placed in the chamber. This time interval corresponded to the first and last 15 min of the IC session, respectively (Figure 1C). Data are presented as the mean distance traveled (cm) ± SEM for the first 15 min of the 1 h session.

### 2.7 Calculations, and statistical analyses

Response ratio and latency data were analyzed using Repeated Measures one-way Analysis of Variance (ANOVA) with Dunnett’s *post-hoc* comparison between each dose and the vehicle control condition. ED_50_ calculations were performed for three primary metrics of the IC task in order to directly compare the relative efficacy of EX4 in disrupting metrics of reward choice (response ratio) and response vigor (nosepoke and reward cup latencies), and to facilitate comparison of the effects to other EX4 studies. For calculations of ED_50_ values, response ratio values were transformed to percent inhibition (% inhibition = [(subject’s vehicle value – subject’s treatment value)/(mean vehicle value) × 100]). As we considered percent inhibition to only be positive, negative values were adjusted to zero. Nosepoke and reward cup entry latencies were transformed to percent maximum effect (% maximum effect = [(subject’s treatment latency – subject’s vehicle latency)/(maximum observed latency in group – subject’s vehicle latency)]). As maximum effects could result in a decrease in latency, we included negative values in these calculations. ED_50_ values were calculated using the least squares linear regression followed by calculation of 95% confidence limits (Bliss, 1967). These transformed data were also analyzed using Repeated Measures one-way ANOVA with Holm-Šidák *post-hoc* tests comparing the effects of each dose. As well, in these transformed analyses, one sample t tests assessed whether treatment group effects differed from vehicle (i.e., change from vehicle as 0%). To assess drug effects during the session, the response ratio, nosepoke latency, number of rewards, and reward latency during the first and last 15 min of the session were compared to the corresponding vehicle condition, and statistically analyzed by a Mixed Model Analysis followed by a Dunnett’s *post-hoc* comparison with vehicle. To assess if EX4 caused a decrease in motivation for the IC from the onset of the session, or if the rat needed to experience the sucrose reward, we quantified which IC the rats first responded to and analyzed this by a Friedman test. Free drinking behavior was analyzed at 2-min and 1-h intervals. 2-min was chosen because the total volume consumed during this period was on average similar to the total consumed during an entire baseline IC session, which was estimated by ([#rewards × 0.06 ml]), (Wakabayashi et al., 2018, 2020). The 1 h interval was chosen in order to compare this behavior within the same duration as the IC session. Free drinking data were statistically analyzed using Mixed Model Analysis followed by Dunnett’s post-hoc comparisons between each dose and vehicle. Locomotor data were analyzed with Mixed Model Analysis followed by a Holm-Šidák *post-hoc* comparison between each treatment and vehicle at both timepoints (2 h and 2:45 h post treatment), and treatment effects between the two timepoints. All statistics were analyzed using GraphPad Prism versions 8–10. For mixed effect model analyses, we used a compound symmetry covariance matrix, fitted using Restricted Maximum Likelihood (REML). For clarity, only significant effects and interactions are reported. The level of significance was set to α = 0.05. As there is some question about the theoretical appropriateness and method of calculating effect sizes on mixed models analyses, estimates of effect size are reported as partial eta-squared (η_p_^2^) only in Repeated Measures ANOVA (Orelien and Edwards, 2008).

## 3 Results

**Figure 2:**
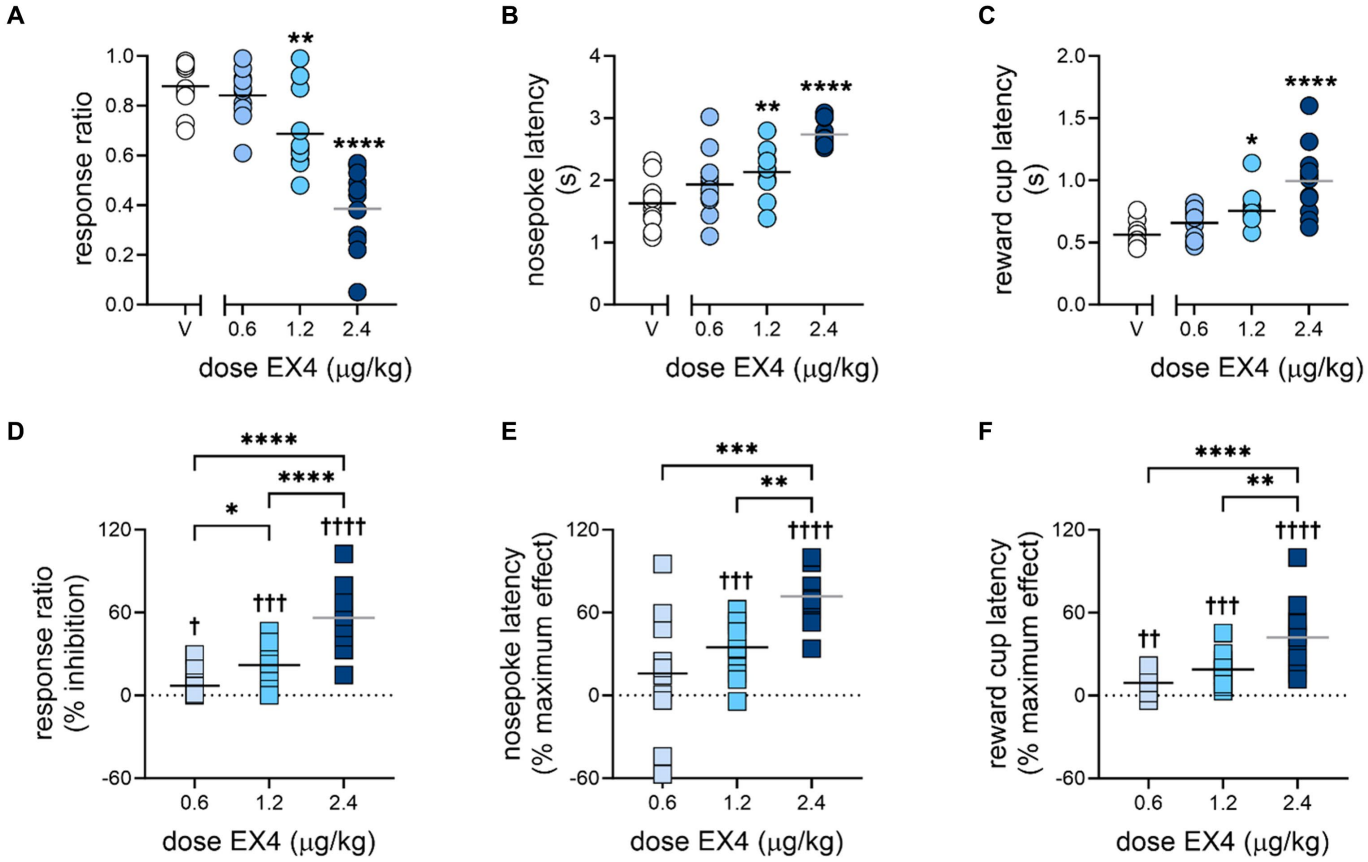
Effects of EX4 on responding during the incentive cue-instrumental task. EX4 dose-dependently decreased the **(A)** response ratio and **(B)** increased the latency to nosepoke and **(C)** enter the reward cup. For generalizability, the same data are transformed as **(D)** % inhibition of EX4 on response ratio, and % maximum effect of EX4 on **(E)** nosepoke and **(F)** reward cup latencies. Bar represents mean, symbols represent individual subjects. *p < 0.05, **p < 0.01, ***p < 0.001, ****p < 0.0001 compared to the vehicle treatment value using the Dunnett’s *post-hoc* test **(A–C)** or to each other using the Holm–Šidák test with brackets indicating the comparison **(D–F)**; †*p* < 0.05, ††*p* < 0.01, †††*p* < 0.001, ††††*p* < 0.0001 using one sample *t*-tests compared to zero, *n* = 11.

EX4 produced a profound dose-dependent decrease in responding to the ICs, along with increases in latency to nosepoke in response to the IC, and latency to enter the reward cup after emitting a rewarded nosepoke (Figure 2, summarized in Table 1). The mean response ratio ± SEM after vehicle was 0.88 ± 0.03, while 0.6, 1.2 and 2.4 μg/kg EX4 produced decreases in the response ratio to 0.84 ± 0.03, 0.69 ± 0.05, and 0.39 ± 0.05, respectively (Figure 2A, Repeated Measures one-way ANOVA *F*_3,30_ = 37.73 *p* < 0.0001, η_p_^2^ = 0.75). When the response ratio was converted to % inhibition, analysis of the dose response determined the ED_50_ to be 2.25 (1.79–2.82) μg/kg. 0.6, 1.2 and 2.4 μg/kg EX4 significantly dose-dependently increased % inhibition of the response ratio (Figure 2D, Repeated Measures one-way ANOVA *F*_3,30_ = 32.38, *p* < 0.0001, η_p_^2^ = 0.75) and from zero [One sample *t-*test 0.6 μg/kg: *t*(10) = 2.42, *p* = 0.036, 1.2 μg/kg: *t*(10) = 4.70, *p* = 0.0008, 2.4 μg/kg: *t*(10) = 7.97, *p* < 0.0001]. The average nosepoke and reward cup latencies also increased. The mean active nosepoke latency increased from 1.63 ± 0.12 s after vehicle to 1.94 ± 0.55, 2.13 ± 0.12, and 2.74 ± 0.06 s after 0.6, 1.2 and 2.4 μg/kg EX4, respectively (Figure 2B, Repeated Measures one-way ANOVA *F*_3,30_ = 18.23, *p* < 0.0001, η_p_^2^ = 0.65). After a % maximum effect transformation, 2.4 μg/kg EX4 significantly differed from 0.6 and 1.2 μg/kg EX4 (Figure 2E, Repeated Measures one-way ANOVA analysis *F*_2,20_ = 11.88, *p* = 0.0004, η_p_^2^ = 0.75) and the middle and high dose of EX4 differed from zero [One sample *t-*test, 1.2 μg/kg: *t*(10) = 5.51, *p* = 0.0003, 2.4 μg/kg: *t*(10) = 11.83, *p* < 0.0001]. Likewise, the mean latency to enter the reward cup after a rewarded nosepoke was 0.56 ± 0.03 s after vehicle, and increased to 0.66 ± 0.04, 0.75 ± 0.05, and 0.99 ± 0.09 s after 0.6, 1.2, and 2.4 μg/kg EX4, respectively (Figure 2C, Repeated Measures one-way ANOVA *F*_3,30_ = 17.75, *p* < 0.0001, η_p_^2^ = 0.80). As a % maximum effect, Repeated Measures one-way ANOVA analysis determined that all three doses of EX4 significantly increased the latency to enter the reward cup from each other and compared to zero [Figure 2F, *F*_2,22_ = 15.08, *p* < 0.0001, η_p_^2^ = 0.874; one sample *t-*test 0.6 μg/kg: *t*(11) = 3.83, *p* = 0.0028, 1.2 μg/kg: *t*(11) = 5.76, *p* = 0.0001, 2.4 μg/kg: *t*(10) = 6.01, *p* < 0.0001]. The ED_50_ of the percent maximum effect in nosepoke latency (Figure 2E) was 1.51 (1.14–1.99) μg/kg EX4 while reward cup latency (Figure 2F) produced an ED_50_ of 3.68 (2.21–6.14) μg/kg EX4.

**Table 1:** Effect of EX4 IC task metrics and free drinking.

**Metrics that significantly differed from vehicle**			
**Dose**	0.6 μg/kg	1.2 μg/kg	2.4 μg/kg
**IC metric**			
Response ratio	⎯	↓	↓
Nosepoke latency	⎯	↑	↑
Reward cup latency	⎯	↑	↑
Total nosepokes	⎯	↓	↓
Nosepoke accuracy	⎯	⎯	⎯
No. of rewards	⎯	↓	↓
Total volume	⎯	↓	↓
Total reward cup entries	⎯	⎯	↓
Cup entries per reward	⎯	⎯	⎯
Active nosepokes per IC	⎯	↓	↓
Inactive nosepokes per IC	⎯	⎯	↓
**Free drinking metrics**			
Volume obtained in 2 min	⎯	⎯	⎯
Volume obtained in 60 min	↓	↓	↓
No. of bouts in 2 min	⎯	⎯	⎯
No. of bouts in 60 min	⎯	⎯	↑
Bout size in 2 min	⎯	⎯	⎯
Bout size in 60 min	⎯	⎯	⎯

A table summarizing the effects of EX4 on IC task metrics and free drinking behaviors. Free drinking behavior include metrics in the first 2 min of consumption (similar to the total volumes obtainable within the IC task) while 60 min resulted in ~5-7X the total volume of sucrose obtainable in the IC task.

**Figure 3:**
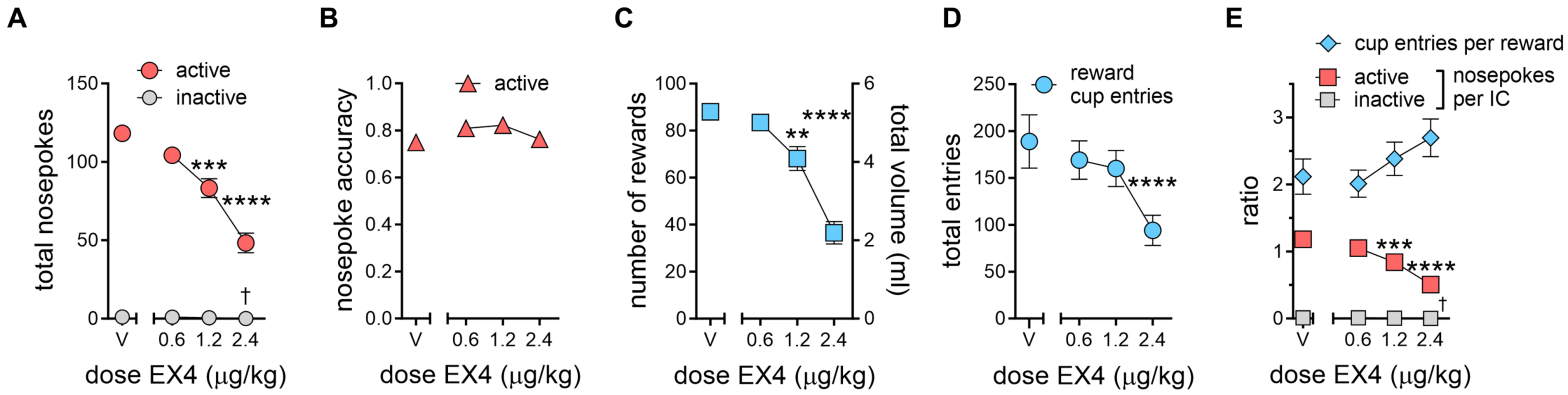
EX4 decreases specific components of IC task performance. **(A)** The total number of active nosepokes during the session dose-dependently decreased. Rats rarely nosepoked in the inactive port, although there was a statistically significant decrease at the highest dose. **(B)** Active nosepoke accuracy was not affected by EX4. **(C)** The number of rewards earned during the session was dose-dependently decreased. The total volume earned is shown on the right *Y* axis. **(D)** The number of total reward cup entries was attenuated by the largest dose of EX4. **(E)** Normalizing responses as a ratio of cup entries per reward (blue diamonds) or active nosepokes per IC (red squares) showed that active nosepokes per IC was dose-dependently decreased, while the ratio of reward cup entries per reward earned trended upward. There was a statistically significant but very modest decrease at 2.4 μg/kg on inactive nosepokes per IC (†, gray squares). *, †*p* < 0.05, **p < 0.01, ***p < 0.001, ****p < 0.0001 compared to the vehicle treatment value using the Dunnett’s *post-hoc* test, *n* = 11.

Rats can emit additional unrewarded nosepokes in the active port either while the reward is being delivered or between IC presentations, leading to more total active nosepokes under vehicle conditions than total ICs presented during the session. EX4 robustly and dose-dependently decreased active nosepokes at each dose. Compared to vehicle pretreatment, which produced a mean of 118.3 ± 5.18 nosepokes per session, 0.6 μg/kg EX4 modestly decreased the mean total active nosepokes to 104.3 ± 4.80, while 1.2 and 2.4 μg/kg EX4 decreased active nosepokes to 83.27 ± 6.02, and 48.27 ± 6.20, respectively (Figure 3A, Repeated Measures one-way ANOVA *F*_3,30_ = 31.82, *p* < 0.0001, η_p_^2^ = 0.76). However, EX4 did not significantly change active nosepoke accuracy, an index that compares rewarded nosepokes to total nosepokes emitted, including those occurring when reward was not available (Figure 3B; vehicle: 0.75 ± 0.03, 0.6 μg/kg: 0.81 ± 0.03, 1.2 μg/kg: 0.82 ± 0.02, 2.4 μg/kg: 0.76 ± 0.02). This indicates that nosepoke response during either the reward delivery or during the non-IC interval was not a significant behavioral factor. In comparison to active nosepokes, rats rarely nosepoked in the inactive nosepoke during the session under any experimental condition (Figure 3A; vehicle: 1 ± 0.381, 0.6 μg/kg: 0.909 ± 0.392, 1.2 μg/kg: 0.546 ± 0.366, 2.4 μg/kg: 0.091 ± 0.091), although this did yield a significant effect of treatment (Repeated Measures one-way ANOVA *F*_3,30_ = 3.069, *p* = 0.043, η_p_^2^ = 0.23).

Examining the number of rewards earned during the IC session, rats achieved 88 ± 3.43 rewards after vehicle. 0.6 μg/kg EX4 did not significantly decrease the number of rewards earned (Figure 3C, 83.5 ± 3.49), while 1.2 and 2.4 μg/kg EX4 both decreased the number of rewards earned compared to vehicle to 68.1 ± 5.12 and 36.6 ± 4.78, respectively (Repeated Measures one-way ANOVA *F*_3,30_ = 39.27, *p* < 0.0001, η_p_^2^ = 0.78). The total number of reward cup entries during the session, a behavioral response with a similar motor demand as nosepokes, was less consistently impacted by EX4 than total active nosepokes (Figure 3D, Repeated Measures one-way ANOVA *F*_3,30_ = 10.67, *p* < 0.0001, η_p_^2^ = 0.52). The mean reward cup entries were 189 ± 28.53 after vehicle, while 0.6, 1.2 and 2.4 μg/kg EX4 decreased this to 169.1 ± 20.57, 160.1 ± 19.31, and 94.09 ± 16.13, respectively. Only the 2.4 μg/kg EX4 dose produced a significant decrease in reward cup entries compared to vehicle.

When considering the number of opportunities to earn a reward during a session, under vehicle conditions rats emitted more nosepokes in the active port than ICs presented, 1.18 ± 0.05 nosepokes per IC (Figure 3E). EX4 dose-dependently decreased this ratio to 1.05 ± 0.05, 0.84 ± 0.06, and 0.51 ± 0.06 after 0.6, 1.2 and 2.4 μg/kg EX4, respectively. Only the two larger doses significantly decreased nosepokes per IC compared to vehicle (Repeated Measures one-way ANOVA *F*_3,30_ = 28.48, *p* < 0.0001, η_p_^2^ = 0.74). This decrease in nosepoke per IC ratio is proportional to the changes in response ratio (Figure 2). A similar analysis on inactive nosepokes per IC revealed a statistical but modest decrease between vehicle and 2.4 μg/kg EX4 (Repeated Measures one-way ANOVA *F*_3,30_ = 3.006, *p* = 0.046, η_p_^2^ = 0.23; vehicle: 0.010 ± 0.004, 0.6 μg/kg: 0.009 ± 0.004, 1.2 μg/kg: 0.005 ± 0.003, 2.4 μg/kg: 0.001 ± 0.001). In experimental terms, this meant that rats nosepoked once in the inactive nosepoke during an IC under vehicle conditions and emitted no inactive nosepokes at the highest EX4 dose. Normalizing the number of reward cup entries to the number of rewards earned during the session revealed that rats entered the reward cup 2.12 ± 0.26 times for each reward earned during the session after vehicle, which did not change after 0.6 μg/kg EX4 (2.02 ± 0.20), but the ratio increased after 1.2 and 2.4 μg/kg EX4 to 2.39 ± 0.25 and 2.70 ± 0.28, respectively (Figure 3E). While the reward cup entries per reward appeared to increase dose-dependently, this did not quite reach statistical significance variance (*p* = 0.08).

**Figure 4:**
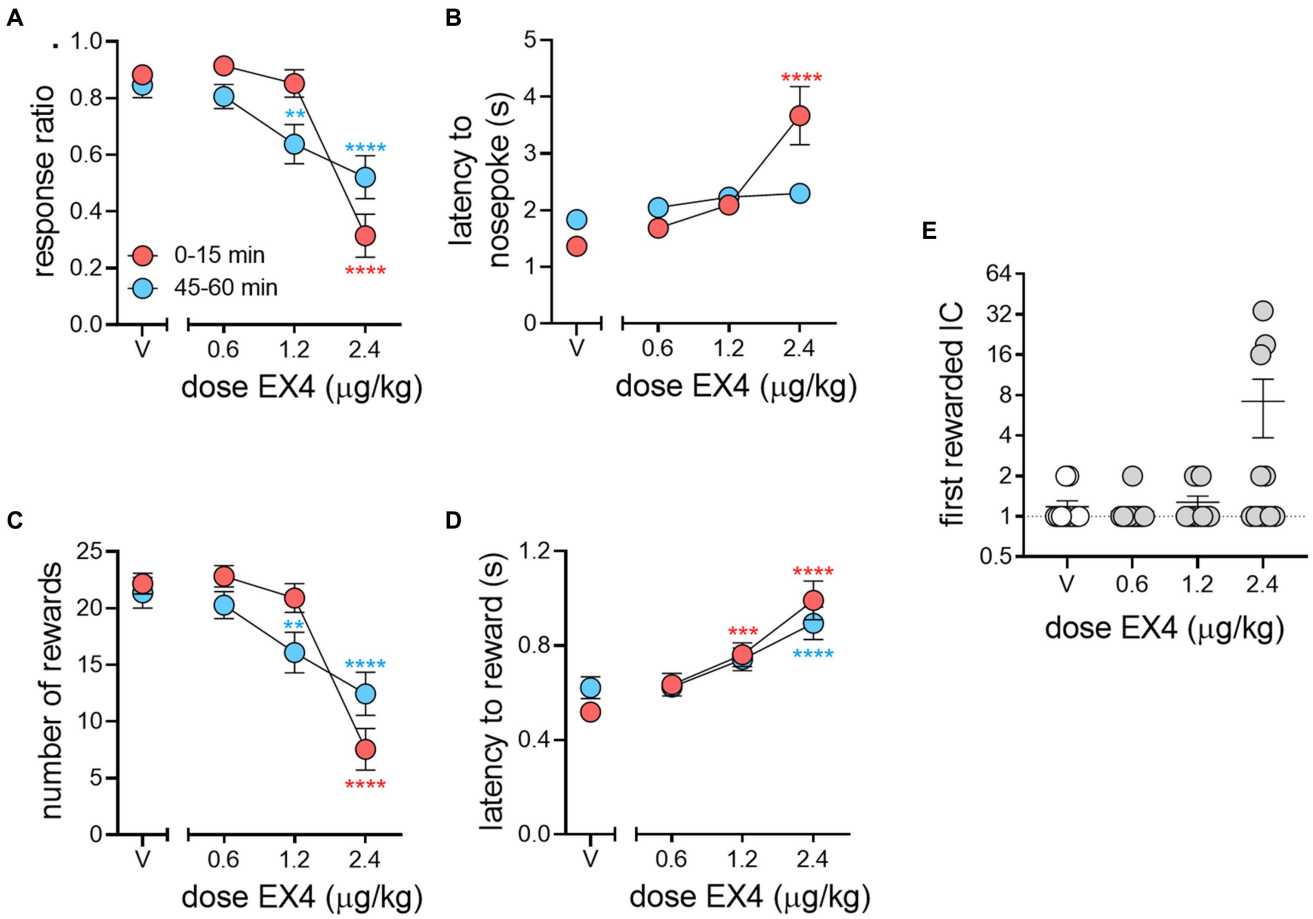
Pharmacokinetic effects of EX4 within an IC session. **(A)** EX4 had dose-dependent effects on response ratio during the beginning (red circles) and end (blue circles) of a session compared to vehicle. **(B)** EX4 dramatically increased the latency to nosepoke during the beginning but not the end of a session at 2.4 μg/kg EX4 compared to vehicle, while **(C)** the number of rewards earned during the session followed similar trends to that of response ratio and **(D)** the latency to reward was greater than vehicle controls throughout the session at the highest dose of EX4, and only during the beginning of the session at the 1.2 μg/kg dose. **(E)** After EX4 treatment, a subset of rats responded to a later IC during the session, although due to group variance, this effect did not reach statistical significance (*p* = 0.06). Circles in **(E)** represent individual subjects. *p < 0.05, **p < 0.01, ***p < 0.001, ****p < 0.0001 comparing dose effects during the beginning or end of session to the same times after vehicle treatment using the Dunnett’s *post-hoc* test, *n* = 11.

We next determined the effect of EX4 over the course of the 1-h session by comparing metrics during the first and last 15 min of the 1-h session to vehicle during the same time periods (results summarized in Table 2). The mean response ratio between the first and last 15-min bin for vehicle were essentially the same at 0.88 ± 0.03 and 0.85 ± 0.04, respectively. The 0.6 μg/kg EX4 dose did not significantly differ compared to controls during these two bins (0.91 ± 0.03 and 0.81 ± 0.04 for first and last 15-min bins, respectively). However, with 1.2 μg/kg EX4 we observed no change in the response ratio in the first 15-min bin (0.85 ± 0.05) compared to vehicle, and a decrease in the last 15-min bin to 0.64 ± 0.07 (1.2 μg/kg). In contrast, 2.4 μg/kg decreased the response ratio in both bins compared to vehicle (first 15 min: 0.32 ± 0.08, last 15 min: 0.52 ± 0.08). We determined there was a significant effect of dose (Figure 4A, Repeated Measures Mixed-Effects Analysis; *F*_3, 30_ = 29.67, *p* < 0.0001) with a significant dose × session bin interaction (*F*_3,30_ = 10.05, *p* < 0.0001), such that a majority of rats only decreased their response ratio near the end of the session after 1.2 μg/kg EX4, but maintained attenuated performance from control throughout the session after 2.4 μg/kg, indicating that the pharmacokinetics of EX4 limited its efficacy at the 1.2 μg/kg dose.

**Table 2:** Effects of EX4 within an IC session.

**Direction of change between first and last 15 min of IC session**			
**Dose**	0.6 μg/kg	1.2 μg/kg	2.4 μg/kg
**IC metric**			
Response ratio	⎯	↓	↑
Nosepoke latency	⎯	⎯	↓
No. of rewards	⎯	↓	↑
Reward cup latency	⎯	⎯	⎯

A table summarizing the effects of EX4 on IC task metrics between the first and last 15 min of the session described in Figure 4.

The mean latencies to nosepoke in response to the IC between the first and last 15-min bins after vehicle were 1.37 ± 0.11 and 1.83 ± 0.17 s, respectively (Figure 4B). EX4, at the two lower doses, did not impact nosepoke latencies during either time points in the session (0.6 μg/kg: 1.69 ± 0.17 and 2.05 ± 0.17, and 1.2 μg/kg: 2.09 ± 0.17 and 2.2 ± 0.16). At the highest dose, this effect was only significantly greater from vehicle at the beginning of the session and dissipated by the end of the session (first 15 min: 3.67 ± 0.51 s, last 15 min: 2.30 ± 0.18 s; Repeated Measures Mixed-Effects Analysis, fixed effect of dose *F*_3,30_ = 13.63, *p* < 0.0001, and a dose × session bin interaction, *F*_3,28_ = 8.11, *p* = 0.0005).

The number of rewards earned from the beginning to end of the session mirrored the pattern in response ratio behavior, with 1.2 and 2.4 μg/kg producing opposing effects. Specifically, the number of rewards after vehicle pretreatment between the beginning and end of the session were 22.18 ± 0.92 and 21.36 ± 1.36, respectively, while 0.6, 1.2 and 2.4 μg/kg EX4 produced 22.82 ± 0.94 and 20.27 ± 1.20, 20.91 ± 1.28 and 16.09 ± 1.79, and 7.55 ± 1.84 and 12.46 ± 1.90 rewards, respectively. Similar to response ratio, analysis of the number of rewards revealed a significant main effect of dose (Figure 4C, Mixed-Effects Analysis; *F*_3,30_ = 31.80 *p* < 0.0001) and a significant dose × session bin interaction (*F*_3,30_ = 8.18, *p* = 0.0004). When compared to vehicle pretreatment, there was no effect of session time at the lowest dose of EX4. At the 1.2 μg/kg dose, the number of rewards were only attenuated during the latter portion of the session. In contrast, at the highest dose, the number of rewards remained attenuated throughout the entirety of the session.

The mean reward cup entry latencies for vehicle between the first and last bins were 0.52 ± 0.03 s and 0.62 ± 0.05 s, while EX4 increased this to 0.64 ± 0.05 s and 0.63 ± 0.04 s (0.6 μg/kg), 0.76 ± 0.05 s and 0.74 ± 0.05 s (1.2 μg/kg), 0.99 ± 0.08 s and 0.89 ± 0.07 s (2.4 μg/kg). There was a significant effect of dose (Figure 4D, Mixed-Effect Analysis; *F*_3,30_ = 19.57, *p* < 0.0001), while the dose × session bin interaction approached but did not achieve significance (*F*_3,28_ = 2.899 *p* = 0.053). Post-hoc analyses revealed that at the beginning of the session, both 1.2 and 2.4 μg/kg EX4 increased reward cup latencies when compared to controls. Conversely, at the end of the session, only rats receiving the highest dose of EX4 demonstrated increased reward latencies. Under control conditions, rats normally responded to either the first or second IC presented in the session. This effect increased in several subjects dramatically at the highest dose of EX4 tested, although this did not quite reach statistical significance because of within group variance (*p* = 0.06).

**Figure 5:**
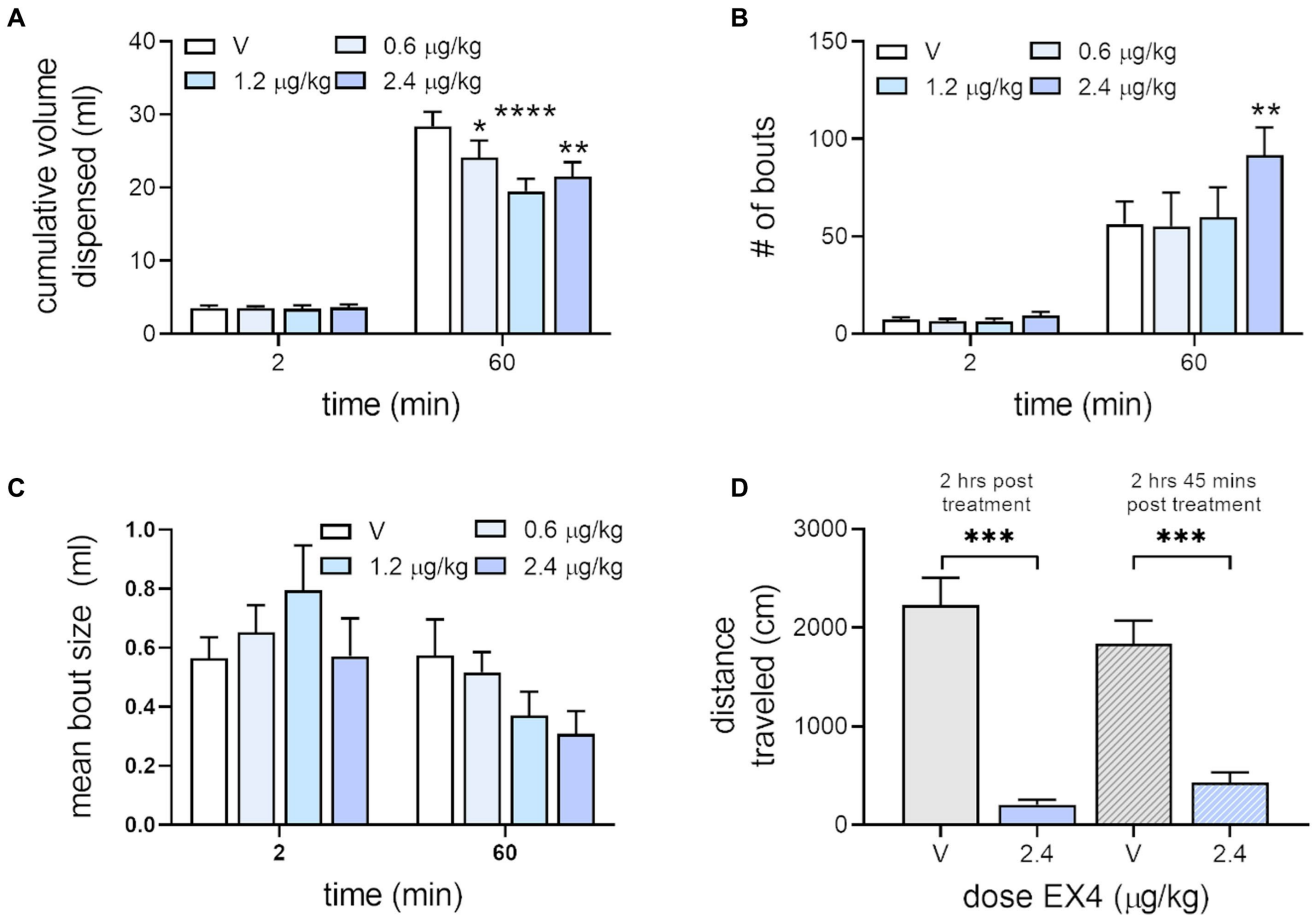
Effect of EX4 on free drinking and locomotion. **(A)** EX4 decreased consumption of reward significantly compared to vehicle after 60 min of free access to sucrose but did not affect consumption within volumes associated with the IC task. **(B)** EX4 increased the number of bouts after 60 min access to sucrose at the highest dose of EX4 tested. **(C)** No statistically significant pattern was seen with average bout size. *p < 0.05, **p < 0.01, ****p < 0.0001, compared to vehicle treatment value using the Dunnett’s *post-hoc* test, *n* = 8. **(D)** While EX4 treatment at both treatment timepoints decreased locomotion compared to vehicle, there were no significant differences between EX4 treatment in either group using the Holm-Šidák *post-hoc* test. ***p < 0.001, brackets indicating the comparison, *n* = 7.

When we examined the effect of EX4 on free drinking of the sucrose reward used in the IC task, we found that the cumulative volume dispensed into the reward cup for vehicle after 2 min, which corresponds to similar total amounts of reward consumed during the IC task (Wakabayashi et al., 2018, 2020) was 3.5 ± 0.33 ml, while EX4 did not change this across the doses tested, 3.56 ± 0.24 ml, 3.41 ± 0.49 ml, and 3.65 ± 0.35 ml after 0.6, 1.2 and 2.4 μg/kg EX4, respectively. In contrast, after 60 min, the total volume dispensed after vehicle was 28.41 ± 1.97 ml, which was decreased by EX4 to 24.14 ± 2.30 ml (0.6 μg/kg), 19.48 ± 1.74 ml (1.2 μg/kg), and 21.53 ± 1.98 ml (2.4 μg/kg). There was a significant effect of session time (Figure 5A, Repeated Measures Mixed-Effect Analysis; *F*_1,7_ = 286.3, *p* < 0.0001), treatment (*F*_3,21_ = 3.58, *p* = 0.311), and a significant dose × session time interaction (*F*_3,21_ = 5.163, *p* = 0.0079). Examining the number of bouts in the free drinking session, we found that at 2 min vehicle treatment produced a mean ± SEM of 7.38 ± 1.07, while EX4 did not change this across the doses tested, 6.5 ± 1.13 (0.6 μg/kg), 6.38 ± 1.46 (1.2 μg/kg), 9.37 ± 1.94 (2.4 μg/kg). However, after 60 min, the number of bouts after vehicle were 56.25 ± 11.64, and after EX4 was dose dependently increased to 55.13 ± 17.36 (0.6 μg/kg), 59.88 ± 15.37 (1.2 μg/kg), 91.62 ± 14.29 (2.4 μg/kg) so that there was a significant effect of session time (Figure 5B, Repeated Measures Mixed-Effect Analysis; *F*_1,7_ = 27.03, *p* = 0.0013) and treatment (*F*_3,21_ = 3.25, *p* = 0.0421). The average bout size after 2 min for vehicle was 0.57 ± 0.71 ml, while EX4 did not change this across the doses tested, 0.65 ± 0.09 (0.6 μg/kg), 0.80 ± 0.15 (1.2 μg/kg), 0.57 ± 0.13 ml (2.4 μg/kg). However, after 60 min, the average bout size after vehicle was 0.58 ± 0.12, and after EX4 was changed to 0.52 ± 0.07 (0.6 μg/kg), 0.37 ± 0.08 (1.2 μg/kg), 0.31 ± 0.08 ml (2.4 μg/kg) so that there was a significant effect of session time (Figure 5C, Repeated Measures Mixed-Effect Analysis; *F*_1,7_ = 16.66, *p* = 0.0047) and a session time × treatment interaction (*F*_3,21_ = 3.44, *p* = 0.0354). Due to variance, no clear statistically significant trends were seen with *post-hoc* analysis.

We also tested rats in locomotor activity chambers to determine if the time-dependent effects seen on operant responding during the beginning and end of the IC session, particularly those seen with 2.4 μg/kg (Figure 4), were due to changes in general activity from the beginning and end of the session (Figure 5D). Our previously published data (Bernosky-Smith et al., 2016) shows that 0.6, 1.2, and 2.4 μg/kg doses of EX4 decreased locomotor activity in the first 5 min of a 60-min session, but only the 2.4 μg/kg EX4 did so in subsequent bins. When we matched the first and last 15 min of the IC task with locomotor sessions timed to begin at equivalent intervals from the EX4 injection (i.e., 2 h or 2 h 45 min post-treatment) we found that 2.4 μg/kg EX4 suppressed locomotor activity in both groups compared to vehicle control (Mixed-Effects Analysis, fixed effect *F*_3, 18_ = 51.08, *p* < 0.0001). Holm-Šidák post-hoc analyses indicated that 2.4 μg/kg EX4 treatments at 2 h or 2 h 45 min post-treatment (equivalent to the timing of the operant behavior analyses in Figure 4) were not significantly different from each other (*p* = 0.2649), indicating that the locomotor effects of the treatment were consistent throughout these two time points. Thus, the general locomotor effects of the highest dose of EX4 used in our study were not significantly different between timepoints matched to the first and last 15 min of the IC and free-drinking session.

## 4 Discussion

In the present study, we determined the effects of systemically administered synthetic EX4 on the ability of ICs to promote reward seeking behavior linked to food. While previous studies have suggested that GLP-1 signaling contributes to consumption, satiety and the motivation to work for food (Dickson et al., 2012; Yang et al., 2014; Bernosky-Smith et al., 2016), it was unclear whether EX4 and other GLP-1R agonists were acting solely via the homeostatic drive for food reward, or through other processes like incentive salience. Here, we show that systemic EX4 dose-dependently attenuated responding to ICs, as well as increased the latencies to respond to both the cues and to enter the reward cup to consume the sucrose reward (Figure 1).

NTS neurons, producing endogenous GLP-1, projecting to the VTA and NAc may modulate the rewarding value of food and other rewards (Merchenthaler et al., 1999; Alhadeff et al., 2012). Studies have shown that systemic and intra-VTA administration of EX4 can attenuate cue-induced cocaine seeking at doses that do not impact food intake (Hernandez et al., 2018). Central administration of EX4 has been shown to attenuate cocaine-induced phasic dopamine signaling in the NAc (Fortin and Roitman, 2017), possibly by increasing GABA_A_ receptor mediated inhibition (Korol et al., 2015a,b; Farkas et al., 2016). Furthermore, Konanur and colleagues have demonstrated that centrally administered EX4 suppresses cue evoked dopamine neuron activity in the VTA during a Pavlovian sucrose seeking task in food restricted rats (Konanur et al., 2020). Our present findings support the hypothesis that systemically administered GLP-1R agonists impact reward-seeking behaviors by disrupting the motivational salience attributed to reward predictive incentive cues.

Others have suggested that operant reward-seeking performance can be parsed into two components quantifying motivation: *response choice*, which represents making a choice between different possible alternative actions, and *response vigor*, which measures the latency with which the chosen action is performed (Niv et al., 2007). In our behavioral task, the choice to enter the active nosepoke or reward cup are quantifiable elements of response choice, while the latency to nosepoke in response to an IC and enter the reward cup after a reward has been delivered, are indices of response vigor. Moreover, to establish the efficacy of EX4 and similar drugs on both response choice and vigor, two aspects of motivation that have not often been dissociated in previous pharmacological studies, we have also determined the ED_50_ of EX4 on choice and vigor metrics for comparisons of potency with EX4 and other drugs in future studies. Determination of the ED_50_ is a foundational principle of pharmacology, yet the ED_50_ of a drug is rarely reported with the IC task. We found that choice-type behaviors more tightly associated with responding to the IC, such as nosepoking in the active port, are robustly attenuated by EX4 (Figures 2A,D, 3A). Likewise, since response to the IC results in the delivery of a discrete sucrose reward under a FR1 schedule, the number of rewards earned is similarly impacted (Figure 3C). However, other less constrained reward-centered behaviors (i.e., the number of reward cup entries) are less attenuated, showing effects only at the highest dose (2.4 μg/kg, Figure 3D). When nosepokes and reward cup entries were normalized to their corresponding events during the session (e.g., the number of ICs presented or rewards delivered, respectively), it appears that behavior associated with motivation for the cues (number of active nosepokes per IC, Figure 3E) were dose-dependently and consistently attenuated, while behavior more associated with motivation for the reward increased (i.e., number of reward cup entries per reward, Figure 3E), although this increase only trended to significance (*p* = 0.06). Importantly, EX4 did not attenuate this reward-related metric at any dose, indicating it did not decrease motivation for the reward. At the same time, EX4 also dose-dependently increased the latencies to respond to the cue with the correct nosepoke (Figures 2B,E) and subsequently enter the reward cup to consume the reward (Figures 2C,F) during the session. This suggests that EX4 also influences vigor-type behaviors present within the IC task as well. Moreover, our ED_50_ analyses suggest that the impact of EX4 differs between vigor-type behaviors with the higher ED_50_, wider 95% confidence interval and rightward shift of the reward cup entry latency dose response indicating that EX4 is more effective in disrupting rat’s response choice and vigor for the IC compared to response vigor for the sucrose itself.

As with our previous studies, we also confirmed that EX4 can affect generalized locomotor activity, however it is important to note that these effects occurred primarily in the first 5 min of the locomotor activity session (i.e., 1 h after EX4 administration) (Mack et al., 2006; Bernosky-Smith et al., 2016). A parsimonious explanation for our results is that EX4 attenuates generalized locomotor activity, including responding in our IC task (Erreger et al., 2012). However, closer analysis of our data suggests a more nuanced effect of EX4. While 0.6 μg/kg EX4 decreased locomotor activity in our previous experiments (Bernosky-Smith et al., 2016), this dose of EX4 had little to no effect on the IC task metrics (i.e., response ratio, nosepoke or reward cup latencies). Further, while EX4 inhibited the total number of nosepokes at the 1.2 and 2.4 μg/kg doses, only the highest dose, 2.4 μg/kg, inhibited reward cup entries (Figure 3), even though both performance metrics require a similar degree of motor activity. Finally, when the locomotor activity test was time-synced to the same treatment timepoints as those of the IC task (Figure 5D), we observed that 2.4 μg/kg EX4 uniformly decreased general locomotor activity at both the beginning and end of the session even though IC task performance was more affected at the beginning of the session than the end. Together this within-session disassociation between locomotion and IC task performance indicates that reductions in IC behavior are not completely correlated to an EX4-induced decrease in overall motor activity.

Closely examining within-session changes in behavior, we found that EX4 had different effects depending on the time of the session and the dose administered when compared to vehicle. For example, we did not observe any significant within-session differences in any IC behavioral metric measured between vehicle and the lowest dose tested (0.6 μg/kg, Figure 4). However, at 1.2 μg/kg, the response ratio and number of rewards obtained were unchanged during the first 15 min but decreased significantly in the last 15 min of the 1-h session. In contrast, vigor-related metrics linked to nosepoking after the IC were not impacted at any point in the session at this middle dose, yet rats took slightly longer to obtain the reward during the beginning but not the end of the session. Therefore, at 1.2 μg/kg, EX4 attenuated IC response choice at the end of the session, while changes in response vigor was limited only towards the reward and was only decreased at the beginning of the session. At the highest dose (2.4 μg/kg), response choice and vigor to obtain the reward were attenuated throughout the session. In contrast, response vigor to the IC, but not IC response choice, appeared to recover by the end of the session. Thus, our data indicate that EX4 affects IC and reward response choice and response vigor differently. This may be attributed to the different dose-dependent pharmacokinetic effects on metrics related to incentive salience while behaviors associated with choosing the reinforcer itself and not the IC (i.e., cup entries and cup entries per reward) remain relatively intact.

A reduction in appetite via an increase in nausea (Anderberg et al., 2016) could also explain our results. We have previously reported that EX4 did not induce pica in a kaolin test at any of the doses reported here (Bernosky-Smith et al., 2016). However, it is possible that the IC task is more sensitive to visceral malaise than the kaolin test, which could explain the decrease in IC responding. Other aspects of task performance disputes this, as the rats entered the reward cup much more frequently per reward at doses that decreased active nosepokes (1.2 and 2.4 μg/kg, Figure 2C), a behavior that seems unlikely if the rats were experiencing nausea. In a free drinking task, EX4 had no effects within volumes of sucrose like those achievable during the IC task (Figure 5A, 2-min bin). EX4 only appeared to have effects when rats were given unrestricted access to sucrose for an hour, indicating an effect on satiety unlikely to be engaged with the smaller, limited volumes attained in the IC task. While 2.4 μg/kg EX4 had an effect on the number of drinking bouts after an hour of free access, a metric that may reflect the potency of post-ingestive inhibition (Davis, 1989; Smith, 2001), there were no corresponding effects at the smaller volumes obtainable during the IC task. Finally, there were no effects of EX4 on the mean bout size at any volume. This implies that nausea, satiety, palatability, or other mechanisms related to post-ingestive homeostatic mechanisms are not major contributors to EX4’s effects on performance in the IC task, particularly considering the much lower volumes of sucrose reward consumed between small discrete rewards and drinking under free access conditions. It should be noted that a lickometer based study showed that EX4 alters sucrose drinking microstructure such that rats drink sucrose less efficiently per bout but compensate for this by drinking more often (Swick et al., 2015). While we did not use lickometers, our observation that EX4 dose-dependently increases reward cup entries could indicate a similar behavioral adaptation. Regardless, future studies directly examining drinking efficiency and microstructure during the IC task would be beneficial.

It has been proposed that changes in incentive motivational processes should occur at the onset of the session, independent of exposure to the reward (Berridge, 2012). In a recent study, centrally administered EX4 dose dependently increased the time to begin licking a sucrose delivery spout that was noncontingently presented immediately after a preceding audio cue in food deprived rats (Konanur et al., 2020). In our study, the rats demonstrated a dose-dependent decrease in motivation for the cue from the onset of the session, as indicated by a decrease in response ratio and nosepoke latency during the first 15 min of the session. Although the rats exhibited a trend to a delay in the first IC they responded to in the task (Figure 4E), this did not reach significance (*p* = 0.06). This effect appears to be independent of hunger or satiety, as our rats were not food restricted during the experiment, in contrast to other studies (Konanur et al., 2020). However, we also observed substantial individual variation in the latency to nosepoke and first IC rewarded at the 2.4 μg/kg dose. This is similar to our previous study in which 2.4 μg/kg EX4 increased the latency to the first lever press in a model of sweetened fat self-administration (Bernosky-Smith et al., 2016). Such individual differences should be explored in future studies of EX4 on motivation.

Our data extends the functionality ascribed to GLP-1 neurotransmission beyond that of nutritive homeostasis, highlighting a possible novel therapeutic strategy to address human disorders involving impaired motivation. Moreover, our current study adds to the growing number studies using an operant reward seeking task (Nicola et al., 2004; Wakabayashi et al., 2004, 2018, 2020; Yun et al., 2004; Ambroggi et al., 2011; Feja et al., 2020) that relies on functioning incentive salience to investigate neurocircuits and systems implicated in reward. The benefits of this and other similar tasks are that the behavior is naturalistic, does not require extensive food or water restriction, is reproducible within an ethically responsible number of animals, and easily adaptable to interrogate different aspects of reward-seeking and reward-taking behavior. Applying ED_50_ analyses on behavioral metrics of this task, as we do here for the first time, is also an important advance, as it increases the task’s generalizability to other drugs and behaviors. Therefore, we view the IC task and its variants as a useful tool to identify likely aspects of reward-seeking and reward-taking behaviors that rely on intact and interrelated processes like incentive salience, reward prediction error, reward sensitivity and motivation (Wakabayashi et al., 2020), that then can be interrogated further. In the context of our study, further investigations into the intersection of incentive salience and GLP-1 would likely be fruitful, and whether these effects are equally represented in rats of both sexes. This is relevant as GLP-1 receptor agonists have sex differences in human weight loss and insulin sensitivity (see Rentzeperi et al., 2022) as well as different effects in male and female rats on the willingness to work for sucrose reward under a progressive ratio schedule (Richard et al., 2016). While female rats have less motivation than male rats to work for sucrose after EX4, and the IC task likely requires similar motivational processes, future experiments will be needed to directly determine the interaction between sex and EX4 on the IC task.

Our findings that synthetic EX4 has dose- and time-dependent effects on distinct components of incentive motivational processes linked to sucrose reward may have clinical implications. For example, continuous intravenous administration of GLP-1 dose dependently increases the feeling of satiety, decreases fullness, and amount of food eaten during a meal or spontaneously in non-obese (Flint et al., 1998) and obese (Näslund et al., 2004) people. Moreover, human functional brain imaging has revealed that continuous administration of synthetic EX4 attenuates brain responses to food cues in reward-related central regions such as the orbital frontal cortex, amygdala, insula, and putamen, but only in obese and not lean individuals (van Bloemendaal et al., 2014). However, considering that therapeutic formulations of synthetic EX4 (e.g., Byetta® or Bydureon®) have distinct pharmacokinetic profiles (Cirincione and Mager, 2017), how the effects of EX4 that we report here impacts the clinical use of GLP-1 therapeutics will need to be further delineated.

In summary, we found that systemic EX4 preferentially attenuates responding to cues attributed with incentive salience. The effects of EX4 on incentive salience were distinct from other components of reward seeking, with the ED_50_ indicating distinct differences in the ability of EX4 to disrupt the reinforcing efficacy of sucrose and the IC. We observed distinct time and dose dependent effects on specific elements of response choice and response vigor towards the IC that could have implications in how synthetic EX4 is prescribed, particularly as used for weight loss and control over hedonic eating habits.

## Data availability statement

The raw data supporting the conclusions of this article will be made available by the authors, without undue reservation.

## Ethics statement

The animal study was approved by University at Buffalo Institutional Animal Care and Use Committee. The study was conducted in accordance with the local legislation and institutional requirements.

## Author contributions

KW: Conceptualization, Data curation, Formal analysis, Investigation, Methodology, Supervision, Validation, Visualization, Writing – original draft, Writing – review & editing. AB: Data curation, Investigation, Validation, Visualization, Writing – review & editing. MF: Data curation, Investigation, Validation, Visualization, Writing – review & editing. MS: Data curation, Validation, Visualization, Writing – review & editing. KC: Investigation, Validation, Writing – review & editing. KB-S: Writing – review & editing. CB: Conceptualization, Funding acquisition, Project administration, Supervision, Visualization, Writing – review & editing.
